# Supplementary material for: PPIFold: a tool for analysis of protein–protein interaction from AlphaPullDown
Source: Bioinform Adv. 2025 Apr 24;5(1):vbaf090. doi: 10.1093/bioadv/vbaf090 (PMC12064169; doi:10.1093/bioadv/vbaf090)
Supplement: vbaf090_Supplementary_Data [file vbaf090_supplementary_data.zip › Rouger_2025_Supplementary-Information_v3.docx]

**SUPPLEMENTARY INFORMATION**

PPIFold: a tool for analysis of Protein-Protein Interaction from AlphaPullDown.

Quentin Rouger, Emmanuel Giudice, Damien F. Meyer, and Kévin Macé

**SUPPLEMENTARY TABLE 1**

The table summarize PPIFold test on R388 T4SS operon proteins in mode all vs all.

| **Protein names** | **UniProt** | **pi_score** | **iptm_ptm** | **pDockQ** | **Result (iQ-score)** | **Result Expected** | **Conclusion** |
| --- | --- | --- | --- | --- | --- | --- | --- |
| **TrwM/TrwK** | O50329_and_O50330 | 1.34 | 0.88 | 0.67 | 76.73 | Yes | Correct |
| **TrwH/TrwF** | O50334_and_O50336 | 2.40 | 0.78 | 0.47 | 75.71 | Yes | Correct |
| **TrwF/TrwE** | O50336_and_O50337 | 1.92 | 0.78 | 0.63 | 76.77 | Yes | Correct |
| **TrwL/TrwI** | O50328_and_O50333 | 1.38 | 0.77 | 0.39 | 65.17 | Yes | Correct |
| **TrwK/TrwD** | O50330_and_O50338 | 2.07 | 0.72 | 0.43 | 70.35 | Yes | Correct |
| **TrwK/TrwG** | O50330_and_O50335 | 0.84 | 0.64 | 0.31 | 55.15 | Yes | Correct |
| **TrwJ/TrwI** | O50331_and_O50333 | 1.81 | 0.45 | 0.23 | 54.17 | Yes | Correct |
| **TrwG/TrwE** | O50335_and_O50337 | 0.77 | 0.46 | 0.22 | 46.31 | Yes | Incorrect (small interface) |
| **TrwL/TrwM** | O50328_and_O50329 | -1.67 | 0.34 | 0.05 | 18.97 | No | Correct |
| **TrwN/TrwL** | Q6I6C7_and_O50328 | 1.69 | 0.46 | 0.17 | 51.72 | Unknown | New interaction or false positive |
| **TrwL/TrwE** | O50328_and_O50337 | -0.73 | 0.42 | 0.02 | 27.46 | No | Correct |
| **TrwN/TrwF** | Q6I6C7_and_O50336 | 1.26 | 0.34 | 0.28 | 48.14 | No | Correct |
| **TrwN/TrwH** | Q6I6C7_and_O50334 | -0.63 | 0.32 | 0.33 | 34.85 | No | Correct |
| **TrwH/TrwG** | O50334_and_O50335 | -1.01 | 0.39 | 0.14 | 28.45 | No | Correct |
| **TrwN/TrwJ** | Q6I6C7_and_O50331 | -0.15 | 0.31 | 0.09 | 30.73 | No | Correct |
| **TrwL/TrwH** | O50328_and_O50334 | -0.16 | 0.21 | 0.03 | 26.13 | No | Correct |
| **TrwE/TrwK** | O50330_and_O50337 | 0.27 | 0.34 | 0.12 | 35.84 | Yes | Incorrect (small interface) |
| **TrwE/TrwI** | O50337_and_O50333 | Bad PAE | Bad PAE | Bad PAE | Bad PAE | Yes | Incorrect (small interface) |
| **TrwN/TrwE** | Q6I6C7_and_O50337 | 0.44 | 0.37 | 0.22 | 41.08 | No | Correct |

**SUPPLEMENTARY TABLE 2**

The table summarize PPIFold test on R388 T4SS operon proteins in mode homo-oligomer.

| **Protein names** | **UniProt** | **pi_score** | **iptm_ptm** | **hiQ-score** | **Result** | **Result expected** | **Conclusion** | **Max Length predicted** |
| --- | --- | --- | --- | --- | --- | --- | --- | --- |
| **TrwN** | Q6I6C7 | 2.08 | 0.69 | 81.41 | 2-mer | 0-mer | New homo-oligomer | 12-mer |
| **TrwL** | O50328 | 0.59 | 0.58 | 59.88 | 5-mer | 5-mer | Correct | 20-mer |
| **TrwM** | O50329 | -2.08 | 0.22 | 15.23 | 0-mer | 0-mer | Correct | 20-mer |
| **TrwK** | O50330 | 0.96 | 0.80 | 72.97 | 2-mer | 2-mer | Correct | 3-mer |
| **TrwJ** | O50331 | 1.38 | 0.88 | 80.90 | 5-mer | 5-mer | Correct | 15-mer |
| **TrwI** | O50333 | 0.47 | 0.71 | 63.72 | 5-mer | 5-mer | Correct | 11-mer |
| **TrwH** | O50334 | No result | No result | No result | 0-mer | 0-mer | Correct | 20-mer |
| **TrwG** | O50335 | 0.38 | 0.58 | 57.59 | 2-mer | 2/4-mer | Correct | 15-mer |
| **TrwF** | O50336 | 2.53 | 0.68 | 85.91 | 3-mer | 14/16-mer | Incorrect (cause of backbone) | 17-mer |
| **TrwE** | O50337 | 1.51 | 0.71 | 75.74 | 10-mer | 14/16-mer | Not enough predicted model | 10-mer |
| **TrwD** | O50338 | 1.27 | 0.67 | 71.38 | 2-mer | 2/6-mer | Correct | 11-mer |

**SUPPLEMENTARY FIGURE 1**


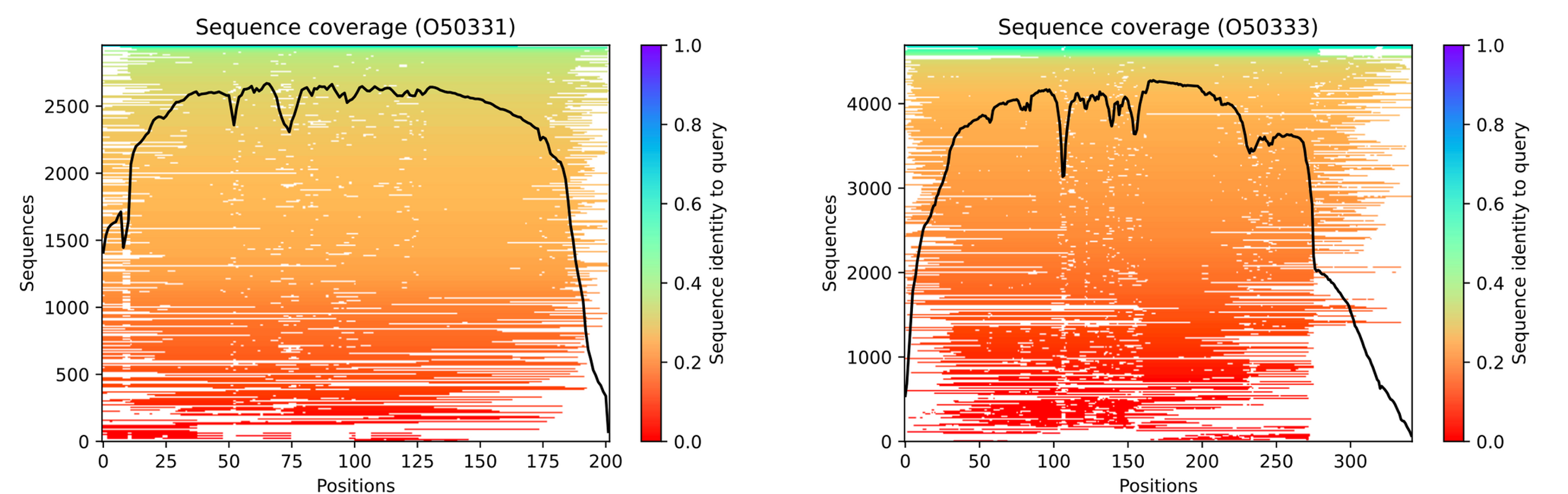


This figure illustrates the depth of the multiple sequence alignment (MSA) for O50331-TrwJ (left) and O50333-TrwI (right), with sequences colour-coded by their level of identity to the reference sequence. The MSA was generated specifically for each protein analysed, providing insights into whether the alignment depth is sufficient to capture co-evolutionary signals essential for predicting both intra- and inter-protein interactions.

**SUPPLEMENTARY FIGURE 2**


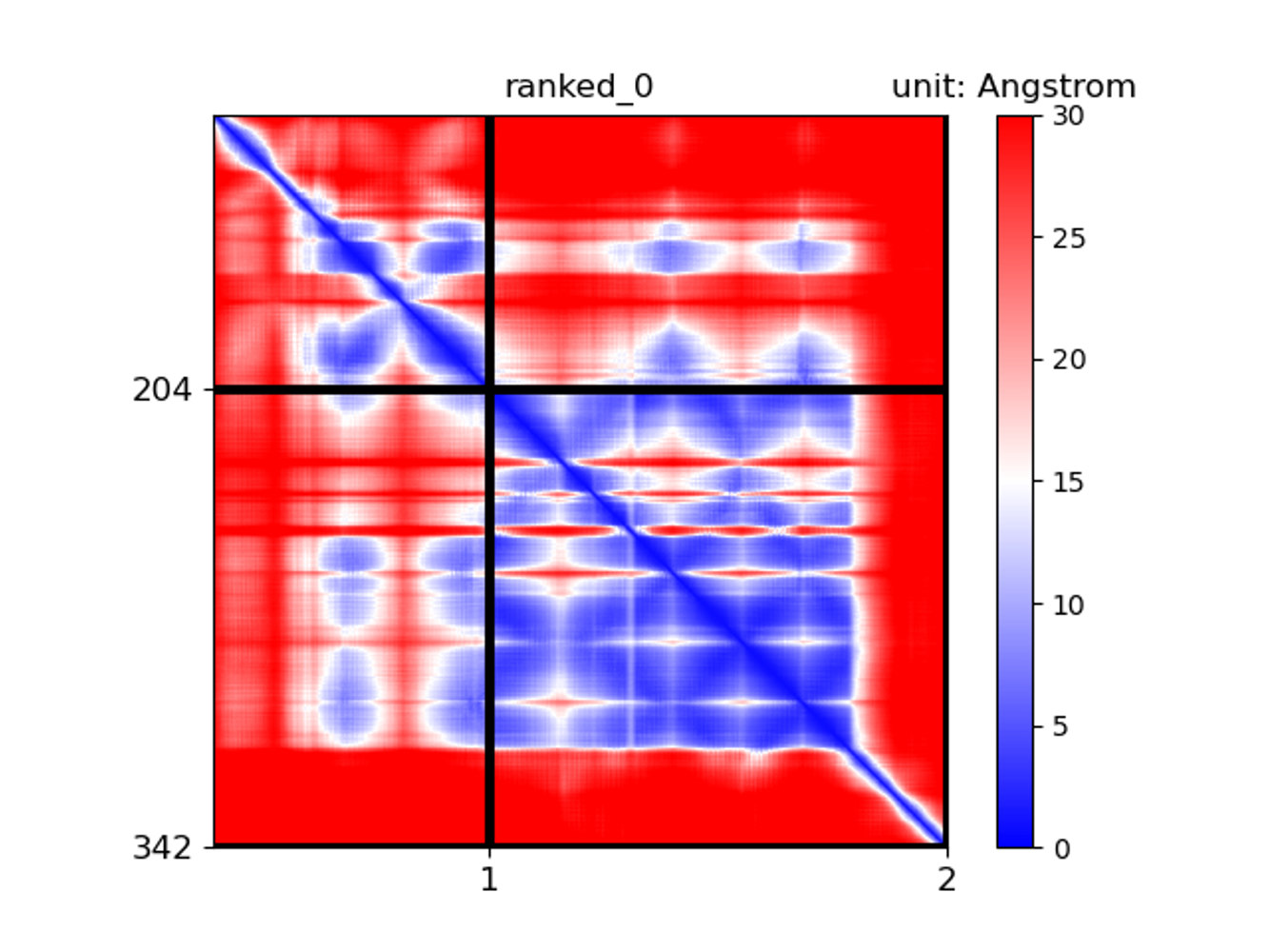


Predicted Alignment Error (PAE) plot for O50331-TrwJ and O50333-TrwI. This figure displays the PAE scores across the proteins, colour-coded based on the error distance in angstroms. Smaller distances indicate higher confidence in the model's predictions.

**SUPPLEMENTARY FIGURE 3**


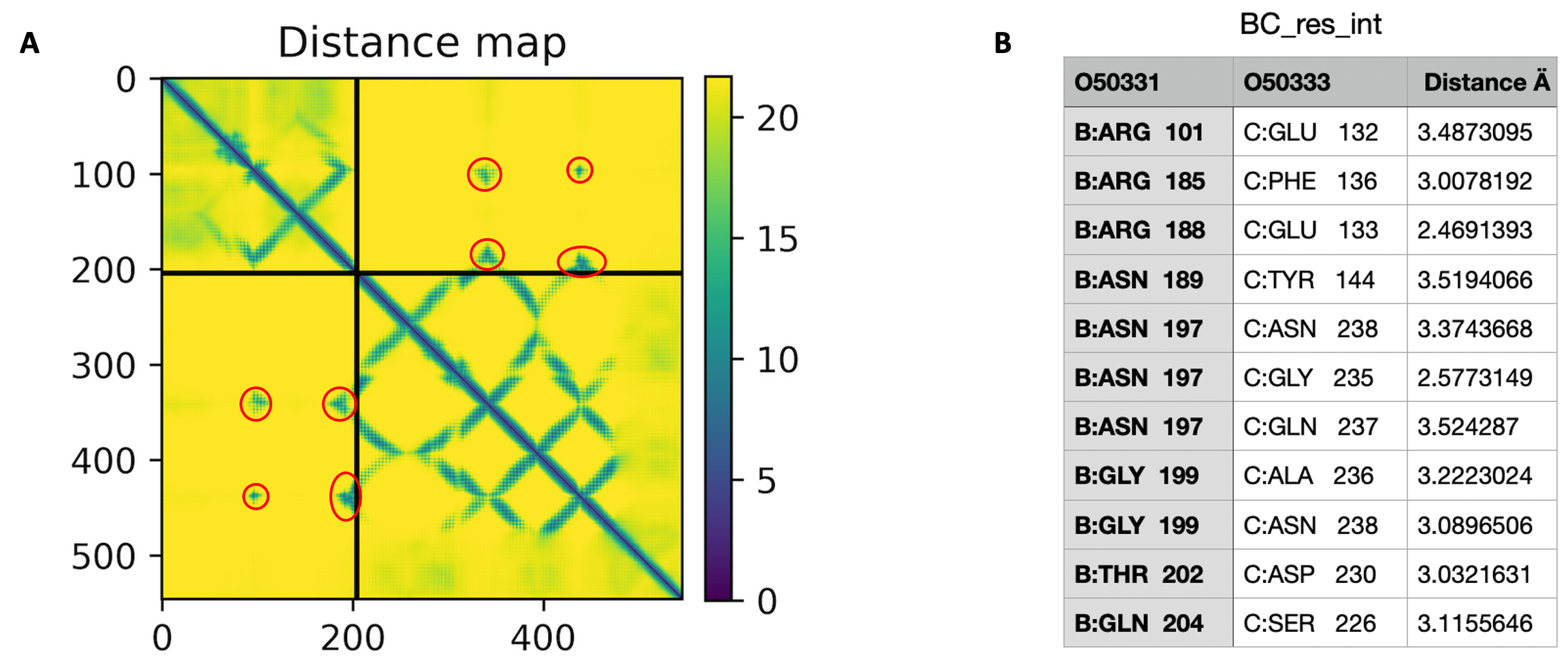


**(A)** The figure presents the distogram for O50331-TrwJ and O50333-TrwI, where the values indicate the protein sequence lengths in amino acids. Points near the diagonal symmetry line and within the black square represent residues in contact within the same protein, while points outside this region, highlighted by red circles, indicate contacts between the two proteins. Colours correspond to the distance between residue pairs in angstroms, with blue points indicating shorter distances. **(B)** The table provides a detailed view of the distogram, focusing on residues in direct contact between O50331-TrwJ and O50333-TrwI (highlighted by red circles in the distogram). This detailed analysis offers insights into specific residue interactions within the model. The table is generated exclusively for the top-ranked protein-protein interactions that satisfy the predefined cutoff criteria.

**SUPPLEMENTARY FIGURE 4**


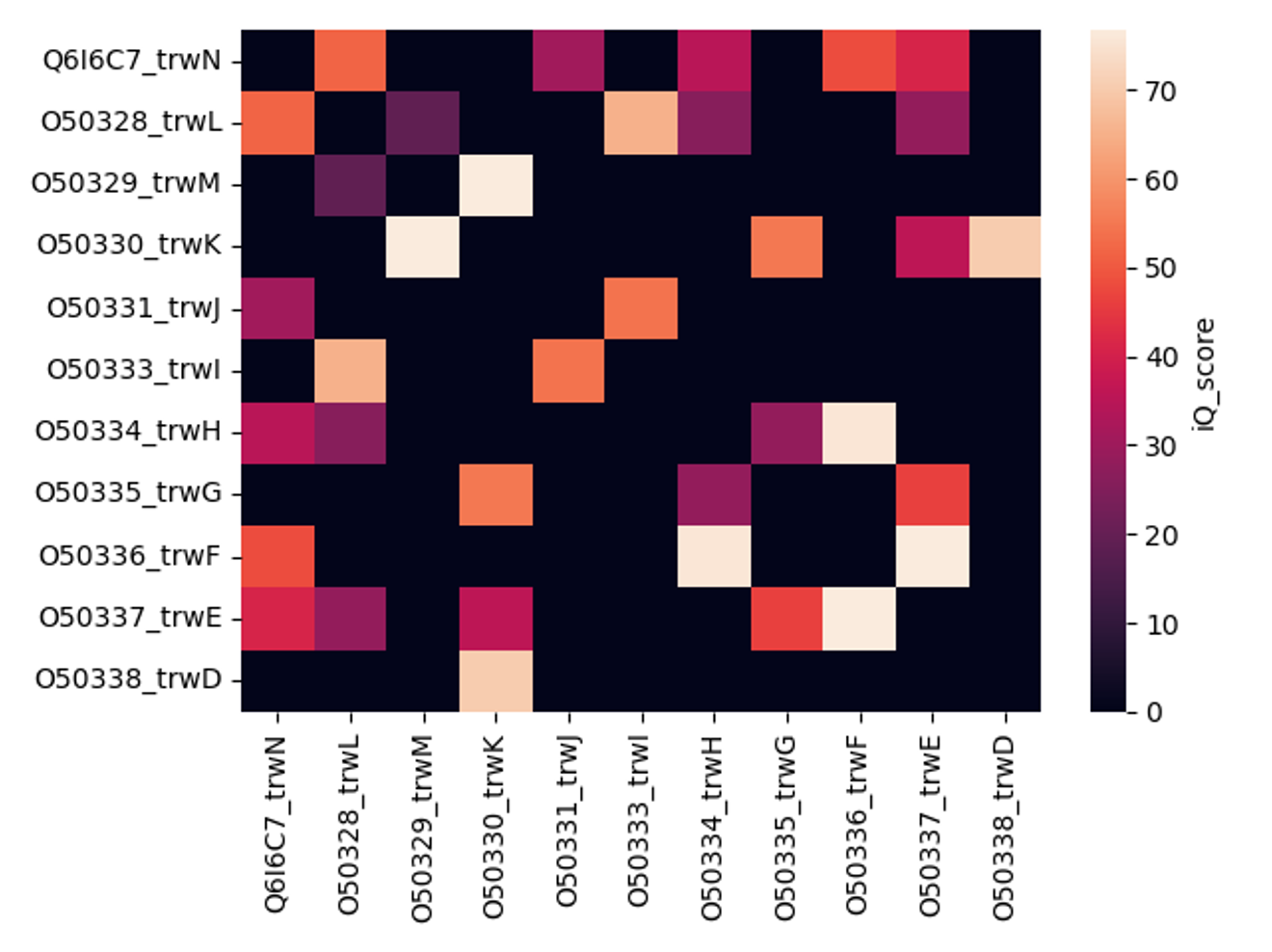


The figure displays a heatmap of interaction scores, where colours represent the iQ-scores. Higher iQ-scores are indicated by lighter colours. Black boxes denote cases with poor PAE scores, homo-oligomerisation, or excessively large total protein lengths. This matrix was constructed using data from Supplementary Data 1.

**SUPPLEMENTARY FIGURE 5**


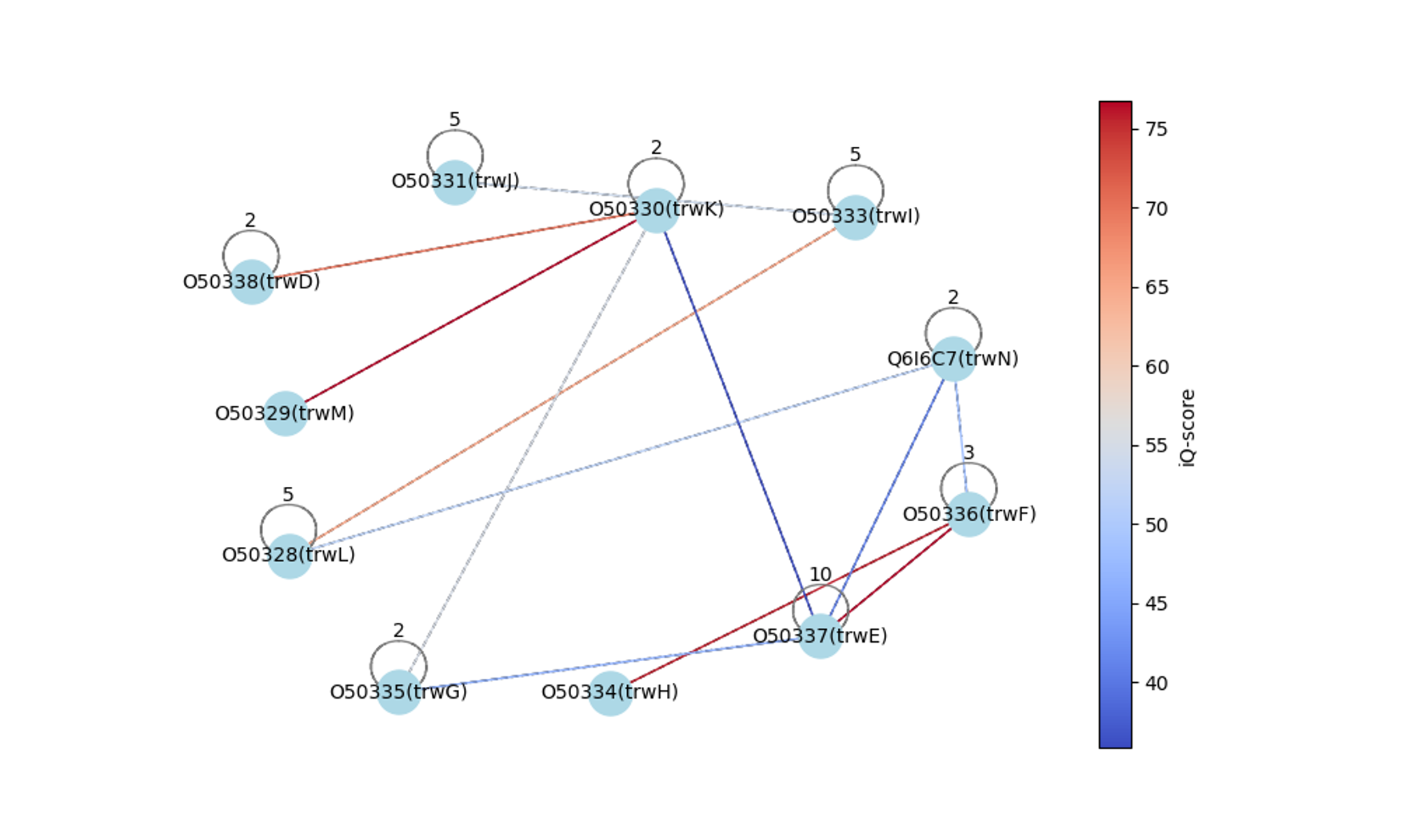


This network represents interactions within R388 proteins and is generated from Supplementary Data 1 and 2. The visualisation includes all interactions that passed the cutoff criteria, as well as homo-oligomer predictions. Each interaction is depicted as a line connecting two proteins, colour-coded according to the corresponding iQ-score. A loop on a protein indicates the most prominent homo-oligomer with the highest hiQ-score.

**SUPPLEMENTARY TABLE 3**

We performed a benchmarking analysis of the iQ-score using publicly available datasets to evaluate its predictive power in protein-protein interaction (PPI) prediction. This benchmark was performed on 50 true positive interactions from the IntAct and MINT databases, and 50 true negative interactions from the Negatome database. The thresholds used are the same as those applied to calculate the iQ-score.

The benchmark includes comparisons to pDockQ, pi_score, and iptm_ptm, focusing on sensitivity and specificity. The iQ-score shows a high sensitivity while maintaining a reasonable level of specificity. It achieves the highest percentage of true positives among predicted positives but has a lower percentage of true negatives than pi_score and iptm_ptm. Notably, the iQ-score is particularly effective at limiting false positives, making it a valuable metric for confident PPI predictions.

| **Score** | **Threshold** | **True Positives (TP)** | **False Negatives (FN)** | **False Positives (FP)** | **True Negatives (TN)** | **Sensitivity** | **Specificity** | **PPV** | **NPV** |
| --- | --- | --- | --- | --- | --- | --- | --- | --- | --- |
| **pi_score** | 0.05 | 39 | 11 | 11 | 39 | 0.78 | 0.78 | 0.78 | 0.78 |
| **iptm_ptm** | 0.5 | 39 | 11 | 10 | 40 | 0.78 | 0.8 | 0.80 | 0.78 |
| **pDockQ** | 0.5 | 24 | 26 | 5 | 45 | 0.48 | 0.9 | 0.83 | 0.63 |
| **iQ-score** | 50.0 | 35 | 15 | 7 | 43 | 0.7 | 0.86 | 0.83 | 0.74 |

The detailed results are provided in two separate Excel files:

**Supplementary Data 1**: True positives and false negatives

**Supplementary Data 2**: True negatives and false positives

**SUPPLEMENTARY FIGURE 6**


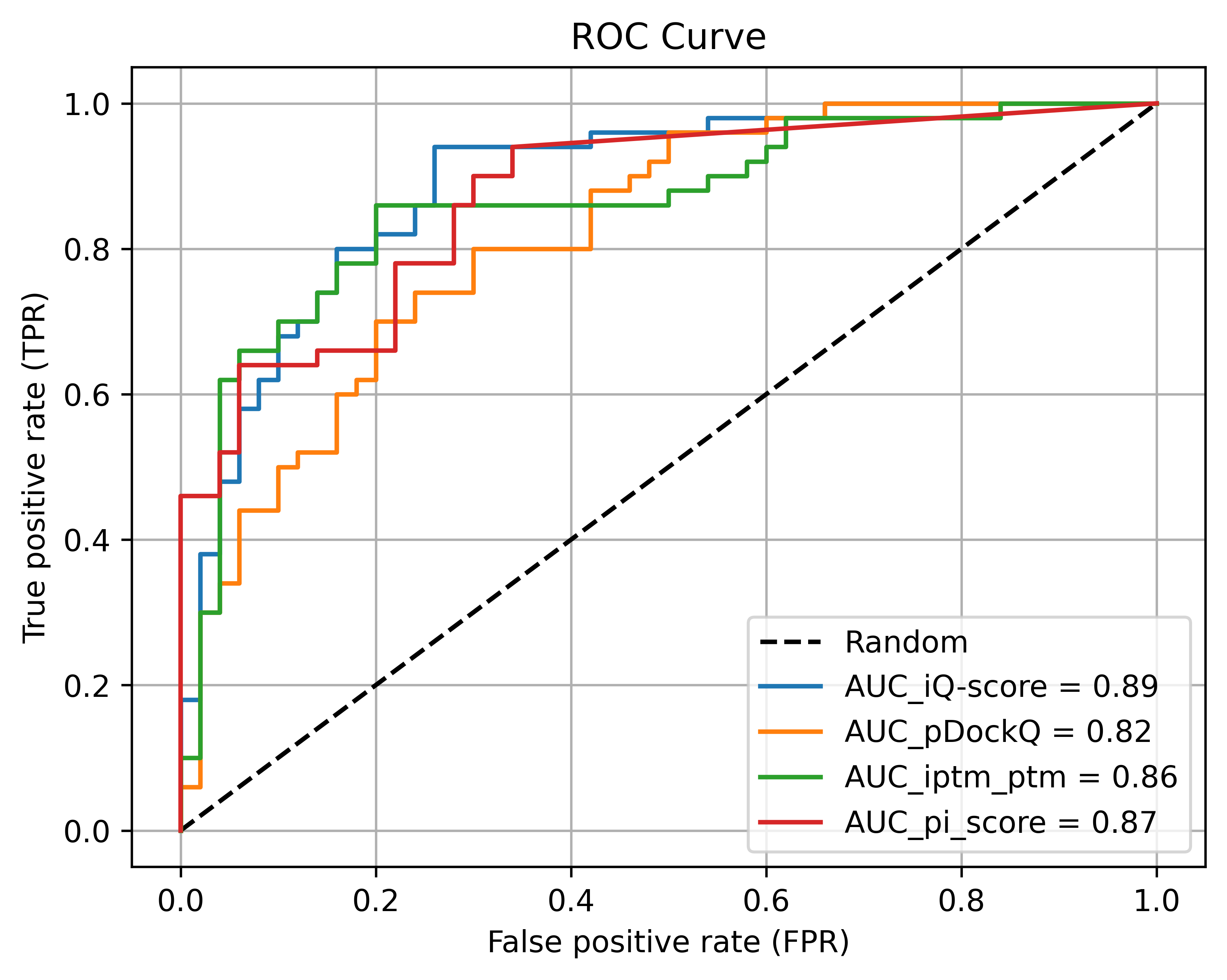


Receiver operating characteristic (ROC) curves and corresponding area under the curve (AUC) values illustrating the predictive performance of iQ-score, pDockQ, ipTM-ptm, and pi-score on the benchmark dataset.
